# Effect of Propolis mouthwash on plaque and gingival indices over fixed orthodontic patients

**DOI:** 10.4317/jced.55026

**Published:** 2019-03-01

**Authors:** Mahboobe Dehghani, Mostafa Abtahi, Nadia Hasanzadeh, Zeinab Farahzad, Mohamad Noori, Meysam Noori

**Affiliations:** 1 Assistant professor of orthodontics, Dental Research Center, Mashhad University of Medical Sciences, Mashhad, Iran; 2 Associate professor of orthodontics, Dental Research Center, Mashhad University of Medical Sciences, Mashhad, Iran; 3 Dental student, Student Research Committee, Mashhad University of Medical Sciences, Mashhad, Iran; 4 Postgraduate student of orthodontics, Orthodontic department, Student Research Committee, Mashhad University of Medical Sciences, Mashhad, Iran; 5 Postgraduate student of orthodontics, Ahvaz Jundishapur University of Medical Sciences, Ahvaz, Iran

## Abstract

**Background:**

In patients with fixed orthodontics, the presence of orthodontic appliances causes dental plaque accumulation and hygiene problems. The purpose of this study was to evaluate the effect of Propolis and chlorhexidine mouthwashes on plaque and gingival indices in patients who are undergoing orthodontic treatment.

**Material and Methods:**

In this triple blind study, in total, 37 patients aged from 15 to 35 years those who have been undergoing fixed orthodontic treatment were studied. After that, one of the mouthwashes that containing either Propolis or Chlorhexidine was randomly prescribed to patients. The patients were asked to use mouthwashes twice a day after brushing their teeth for three weeks consecutively. Indicators of plaque, gingival and periodontal status (PI, GI, CPI) were determined on Ramford teeth at the beginning and at the end of three weeks for each patient. Then the results were analyzed statistically.

**Results:**

The difference between the values of plaque index (*P*<0.001), gingival index (*P*=0.006) and periodontal index (*P*= 0.005) before and after administration of Propolis were statistically significant. The difference was also statistically significant for all three indexes of plaque (*P*<0.001), gingival (*P*=0.001) and periodontal (*P*=0.003) before and after chlorhexidine mouthwash usage. The indices after using mouthwashes were not statistically significant different between two mouthwash groups.

**Conclusions:**

It seems that Propolis mouthwash can be used as a suitable alternative in patients with fixed orthodontic treatment without the side effects of chlorhexidine mouthwash.

** Key words:**Mouthwash, antimicrobial, Oral hygiene, Propolis, Dental plaque.

## Introduction

In fixed orthodontic patients, the presence of malocclusion and crowding with the presence of orthodontic appliances causes hygienic problems. So that the presence of brackets, elastics, wires and other parts of the orthodontic appliances in the mouth causes the pH and bacterial flora of the mouth to change and dental plaque accumulation that is hard to clean (1,2).

Dental plaque is a white to yellowish or grayish substance that creates a strong bond with the surface of the teeth or other hard surfaces in the mouth. These plaques are essentially bio-films of gram-positive and gram-negative bacteria (3) which their metabolites causing inflammation of the gingival and periodontal structures, and dental caries (4). Most people may not have done enough mechanical removal of the plaque, or if these are done, they are not enough to prevent periodontal disease and plaque accumulation (4,5). Therefore, daily oral washing with antimicrobial agents improves oral health and can act as an effective way to control and eliminate bacterial plaques and limit gingivitis and periodontitis (4). Types of toothpastes, mouthwashes and gels are among those antimicrobials whose effectiveness has already been proven to reduce the microbial load of the mouth (6-8).

In recent decades, Propolis has been considered as a medicine. Propolis is made by honey bees, and generally its compounds have included, 50% resin with the plant origin, 30% wax, 10% aromatic and essential oils, 5% pollen and 5% other contains. Of course, the ingredients and chemical composition of Propolis are very diverse regarding to weather, season and area (9). The antimicrobial and therapeutic properties of this substance have paved the way of utilization of this substance in oral hygiene. Therefore, to prove the medicinal effects of Propolis, numerous scientific researchers have been conducted on this subject. The meta-analyzes performed by Yueh-Juen *et al.* with the aim of examining the effects of Propolis on oral hygiene showed that although Propolis can reduce dental plaque, this effect is not statistically significant. Hwu Y-J and Lin F-Y showed that the effect of this substance on oral infection or stomatitis is also not significant (10). In a study by Koo *et al.* that have been done with the aim of evaluating the effect of Propolis mouthwash on 3-day dental plaques, the results showed that plaque index was significantly less in the experimental group than in the control group (11).

The limited clinical examinations on the effect of this substance on plaque and gingival status, and the contradictory results of available investigations, and the significance of plaque control in orthodontic patients, were the indispensable reasons has prompted to perform this clinical research. The purpose of this study was to assess the effect of Propolis mouthwash on plaque and gingival indices in patients undergoing orthodontic treatment and comparison with chlorhexidine mouthwash as Gold standard.

## Material and Methods

-Study design and population:

This research was conducted in 2016, at the orthodontic clinic of Mashhad dental school as a prospective randomized study on subjects under fixed orthodontic treatment. The study was a triple-blind parallel-group clinical trial. The research protocol was approved by the Ethical Committee and the Research Deputy of the Mashhad University of Medical Sciences, Mashhad, Iran (Ethical code: IR.MUMS.sd.REC.1394.83(. Using Dodwad *et al.* study (12) and comparing the means of two independent samples with a power of 80% and error rate of 5%, sample size was determined as 17 patients in each group. Considering the probability of patient dropout, sample size in each group was increased to 20.

In initial step, after explaining the research aims, process, and the benefits and side effects of mouthwash, informed consent was taken from patients.

Inclusion criteria were: good health status, presence of fixed orthodontic appliances, age range of 15-35 years, presence of mild to moderate gingivitis, and complete patient satisfaction. Exclusion criterion were pregnancy or lactation, smoking, diabetes, periodontal disease, severe gingivitis, antibiotic use over the past 2 weeks, history of using mouthwash in the last month, and history of increased sensitivity to Propolis or honey combinations. After considering these criteria, 40 patients treated by one orthodontist were selected and enrolled in the study. In the next step, the demographic data including age, gender, birth date, and orthodontic starting date were obtained using a self-reported questionnaire. Two tables of random numbers, one for the male population and one for the female population were used and the subjects were thus randomly assigned to one of two treatment groups (n=20 in each group). One group, received Propolis aqueous extract and the other group received chlorhexidine mouthwash. Then, plaque, gingival and periodontal status indices (PI, GI, and CPI respectively) were determined on Ramford teeth (16,21,24,36,41,44)and the data were recorded in special forms. For plaque and gingival indices, the Silness & Loe (1964) criterion and for periodontal index (13), the World Health Organization (WHO) (1994) criterion were used. Scores were taken from all participants by the same blinded trained examiner at baseline and 22th day. The examiner was a dental student. Before the study the examiner was calibrated in the use of indices by an experienced periodontist.

In this study, two types of mouthwashes were prepared and encoded in similar bottles. The coding was done by a person outside the study so that both the patients and the investigator of the program and also the statistician, would remain blind about the coding.

-How to prepare mouthwashes:

•Propolis Mouthwash.

30 g of Propolis was combined with 100 ml of distilled water and then mixed with a mixer at 30 ° C for 2 hours. After centrifuging the resulting mixture, the aqueous extract of the Propolis was purified by 30% as the base concentration. Ultimately, the mixing solution of Propolis was 1% with a salt concentration of 0.25%, and along with the essential oil of saffron and flavor, a mouthwash solution was prepared. The prepared solution was poured into 60 same bottles.

•Chlorhexidine mouthwash:

0.2% chlorhexidine mouthwashes were mixed with distilled water with proportion of 3 parts mouthwash and 2 parts water, to obtain a 0.12% chlorhexidine solution. Then was poured into 60 bottles of the same type as the bottles used for the Propolis mouthwash.

-How to use mouthwashes:

The participants of the study were asked to use the assigned mouthwash twice a day (morning and evening) after brushing for three weeks consecutively. They were instructed to rinse with 15 mL of the mouthwash for 1 minute followed by expectoration of the residual mouthwash and then avoid eating and drinking till 30 minutes and not using another mouthwash during the study period. Since each bottle contained 250 mL mouthwash, three bottles of the same mouthwash were given to each patient. The protocol of the use of mouthwash was also provided to patients in written form. To avoid the effect of new variables, subjects were asked to continue their usual daily brushing method (tooth brush and dental floss) during the study period.

Patients were re-examined after three weeks of using mouthwash to re-evaluate the above mentioned indices (plaque, gingival and periodontal indices) on Ramford teeth. The data were recorded to compare the conditions between pre and post mouthwash use.

-Statistical analysis:

In the descriptive statistics section, mean and standard deviation were reported for quantitative variables and percent and number for qualitative variables. Kolmogorov-Smirnov test was used to test the distribution of quantitative data. In the analytic statistics section, according to the data distribution as well as data type, Kruskal-Wallis statistical tests, variance analysis, paired t-test and Wilcoxon test were used. The level of significance was *P*<0.05. Finally, after the statistical analysis, the codes were broken and the results were interpreted.

## Results

40 patients were qualified to be included in the study. Since three persons were dropped out during the following, the final number of samples in the propolis and chlorhexidine groups were 18 and 19, respectively. Among these, 10 were men (27.03%) and 27 women (72.97%). The average age of participants was 19.86 ± 4.19. The descriptive results showed that the average time since start of orthodontic treatment in propolis and chlorhexidine groups was 17.8±9.7 and 19.8±12.6 months, respectively.  Table 1 shows the demographic information of participants.

**Table 1:**
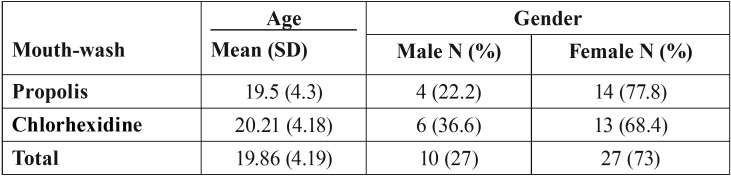
Demographic characteristics of participants in two groups of the study.

Individual reports of participants about the test of mouthwashes showed that for at least five persons Propolis had a desirable taste while for one person the taste was undesirable. For chlorhexidine, six persons reported a spicy taste and one among them had undesirable feeling. In terms of staining of the teeth, about seven of those who were consuming chlorhexidine complained about brown staining of their teeth. However among them, 2 were dropped out from the study. For Propolis mouthwash, one person reported whither teeth, and one reported decrease of calculus from mandibular central teeth, so that she wanted to know the trade name of the mouthwash.

Kruskal–Wallis test showed that in the first visit (baseline measurements) the Plaque Index (PI), did not have any significant difference (*P*=0.474) between two groups. At the second visit (final measurements), also, variance analysis test did not show any significant different in PI (*P*=0.764) between two groups. Based on the results of Kruskal-Wallis test, difference in PI for the first and second visits, between two mouthwashes was not statistically significant (*P*=0.506) ( Table 2).

**Table 2:**
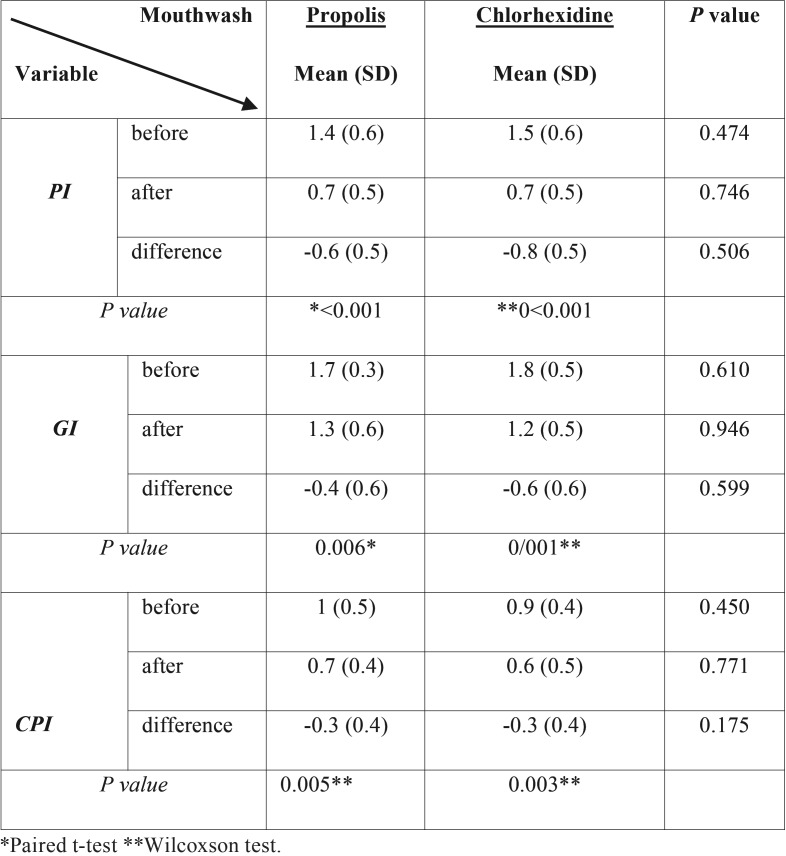
Plaque index (PI), Gingival index (GI) and Periodontal index (CPI) values before and after using mouthwashes.

For GI and CPI, the Kruskal-Wallis test also showed that at the first visit no significant difference between mouthwashes was observed ( Table 2). In addition, comparing these two indicators for the second visit as well as comparing the difference between the first and second visits using variance analysis test, did not show any significant difference between two mouthwash groups.

**Figure 1:**
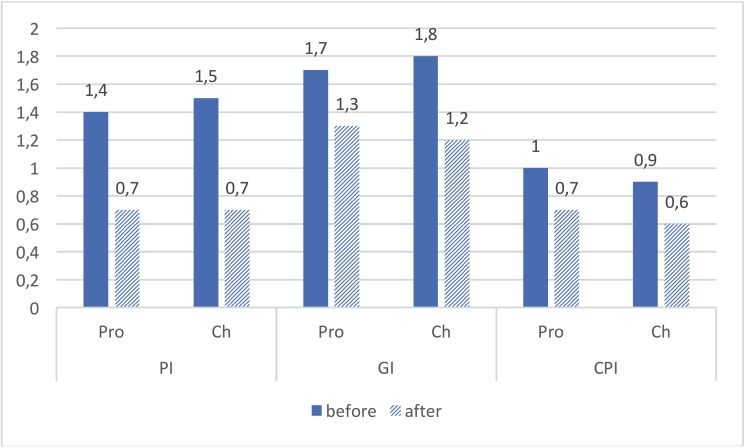
Mean values of Plaque index (PI), Gingival index (GI) and Periodontal index (CPI), before and after using Propolis (Pro) and Chlorhexidine (Ch) mouthwashes.

Also, Paired T-test analysis showed that the observed differences in PI, GI, and CPI indices before and after using propolis mouthwash were statistically significant. Also, a significant difference was observed for the three indices before and after using chlorhexidine mouthwash.  Table 2 and Figure 1 show the data on three mentioned indicators, for both groups.

## Discussion

The current study investigated the effect of Propolis mouthwash on the gingival health of individuals who are had orthodontic fixed appliances. The results showed that both Propolis and chlorhexidine mouthwashes created significant improvement of three investigated gingival and periodontal health indices.

The findings of the current study are consistent with some of previous studies. Anauate-Neotto *et al.* investigated the effect of Propolis and chlorhexidine mouthwashes on the gingival health. Their results showed that Propolis mouthwash significantly reduces the Papillary Bleeding Index. Also, comparing the younger patients with olds showed that the Propolis is more effective in younger ones than olders (14). The current study is consistent with Anauate-Neotto *et al.*, so that changes in the GI and CPI before-and-after using propolis and chlorhexidine mouthwashes was statistically significant and using mouthwash reduced both of them.

Tanasiewicz *et al.* study on the effect of a toothpaste and a gel containing 3% Propolis on the status of tooth cavities, also confirm the findings of the current study. Their results showed that using hygiene products containing Propolis, either in healthy people (in terms of periodontitis) or individuals with gingivitis is effective and remove the plaques and improve the marginal periodontium (15).

The current study also reached the same results, which Propolis mouthwash is effective in orthodontic patients that because of brackets and therefore inability to achieve sufficient oral hygiene are susceptible to oral disease. So that using Propolis resulted in reduction of CPI and PI by 30 and 60%, respectively. As well, Pereira *et al.* in a study on the effect of free alcohol mouthwash, consisting of 5% green Brazilian Propolis, in controlling plaques and gingivitis, found that it significantly reduces the PI and GI by 24 and 40%, respectively, in comparison with the beginning of the study (16). We also found significant reductions in PI (60%) and GI (40%). However, our findings are not consistent with some other studies. A meta-analysis by Yueh-Juen *et al.* found that Propolis reduces the plaque but it was not statistically significant. It seems that the main reason of different findings is applying different protocols, so that in 8 studies that were included in the mate-analysis either the consumption duration or frequency as well as concentration of the mouthwash was less than its equivalent in the current study (10). Generally, since the Chlorhexidine is the gold standard of studies which investigate the effect of mouthwashes (17,18), based on the findings of the current study that showed no significant difference between three evaluated oral health indices (i.e. PI, GI and CPI) after using mouthwash, it can be noted that the propolis mouthwash has almost the same outcomes of Chlorhexidine.

To justify, in addition to antibacterial features of the propolis there is another assumption: “mechanical effects of oral irrigators”. As York *et al.* study on the effect of irrigation with water on periodontal status of fixed orthodontic patients, the control area had more plaque than the area that was irrigated (19). Cutler *et al.* investigated the effect of oral irrigation on periodontitis. They found that it reduces probing pocket depths, bleeding on probing, GI, and PI. They also found that it reduces IL-B levels up to 7 days and PGEs up to 14 days (20). The promising results of Propolis mouthwash in current study maybe attributed to the mechanical effect of irrigation. Since there was not a negative control group, we could not judge with certainty.

With regard to the dissatisfaction among those who were using chlorhexidine, because of its spicy taste and browning of teeth, in comparison with relative satisfaction from Propolis mouthwash, it can be stated that Propolis is an appropriate alternative for chlorhexidine. An alternative that have not the side effects of chlorhexidine.

Finally, it is suggested to increase the sample size in the future studies. So without using non-parametric tests more precise interpretations can be made. Also, to make interpretations about long-term effects of the mouthwash, and in different time periods, it is suggested to increase the duration of mouthwash consumption in future investigations.

As conclusion, considering the limitations of current study, it seems that using Propolis mouthwash has a desirable effect on gingival health of patients with fixed orthodontic appliances. So that it improved the gingival index, plaque index and community periodontal index and its effect was comparable to chlorhexidine. Based on the findings, the authors recommend regular use of Propolis mouthwash in fixed orthodontic patients. As well, based on the findings no side effects were reported.
